# Comparative effectiveness of intravenous versus inhalational antibiotic administration for the management of Acinetobacter pneumonia: A randomized controlled trial

**DOI:** 10.5339/qmj.2026.54

**Published:** 2026-08-18

**Authors:** Kostiantyn Krenov, Oleksandr Oliynyk, Maryna Mamonova, Svitlana Yaroslavska, Andriy Dobrorodniy

**Affiliations:** 1 Department of Surgery with a Course in the Basics of Dentistry, Vinnytsia National Medical University named after M.I. Pirogov, Khmelnytsky, Ukraine; 2 Department of Emergency Medicine and Intensive Care, University of Rzeszow, Rzeszow, Poland; 3 Department of Anaesthesiology and Intensive Care, Bogomolets National Medical University, Kyiv, Ukraine; 4 Department of Anaesthesiology and Intensive Care, I. Horbachevsky Ternopil National Medical University, Ternopil, Ukraine

**Keywords:** Ventilator-associated pneumonia, colistin, procalcitonin, creatinine, logistic regression, sepsis

## Abstract

**Background:**

Aerosol antibiotics directly target the respiratory tract, bypassing the poor lung penetration of many intravenous antibiotics. However, they seem like they could be useful for treating ventilator-associated pneumonia (VAP); not enough research has been done on how well inhaled and intravenous treatments work. The existing literature does not give definitive information regarding which method yields superior therapeutic outcomes.

**Objective:**

To evaluate the therapeutic effects of intravenous versus inhaled colistin in treating *Acinetobacter* VAP in severe traumatic brain injury patients.

**Methodology:**

This prospective, multicenter, randomized, double-blind study compared inhaled versus intravenous colistin for *Acinetobacter* VAP using clinical and laboratory markers of infection and organ function. It was conducted from February 1, 2023, to March 1, 2024, at four Ukrainian medical institutions in Kyiv, Ternopil, and Khmelnytsky.

**Results:**

Both routes significantly reduced leukocyte counts (*p* < 0.001) and clinical pulmonary infection score (CPIS) (*p* < 0.001). Inhaled colistin improved PaO_2_/FiO_2_ ratios from 337 to 343 mmHg (*p* = 0.010), while intravenous colistin did not show a significant change (*p* = 0.334). Intravenous administration better preserved systemic organ function, with sequential organ failure assessment (SOFA) scores worsening from 12 to 13 (*p* < 0.001), compared to inhalation, which worsened from 12 to 14 (*p* < 0.001). Neither route significantly affected sepsis incidence (inhalation 33% vs. intravenous 22.5%, *p* = 0.116) or 28-day mortality (inhalation 27% vs. intravenous 20.6%, *p* = 0.362). Male gender was an independent predictor of mortality (OR 0.44, 95% CI 0.20-0.99, *p* = 0.046).

**Conclusion:**

Post-treatment physiological status was the primary determinant of outcomes in *Acinetobacter baumannii* VAP patients with severe traumatic brain injury.

## 1. INTRODUCTION

*Acinetobacter baumannii* is a Gram-negative coccobacillus that is widely distributed in the environment and commonly found in soil, water, on skin, and in food.^1^ It has emerged as one of the most prevalent pathogens in hospital-acquired infections, particularly in intensive care units.^2^ It can cause meningitis, urinary tract infections, sepsis, skin and soft tissue infections, and, most notably, ventilator-associated pneumonia (VAP). VAP is defined as pneumonia developing after at least 48 hours of mechanical ventilation. This condition is associated with increased mortality, prolonged hospitalization, and higher treatment costs.^3^

The most common forms of ventilator-associated pneumonia (VAP) are caused by such bacterial pathogens as Pseudomonas aeruginosa (20–25%), Staphylococcus aureus (15–20%), Klebsiella pneumoniae (10–15%), and Acinetobacter baumannii (10–15%).^4^ Among these, VAP caused by *Acinetobacter baumannii* is considered one of the most severe forms of hospital-acquired infections, accompanied by high mortality rates and significant treatment costs. According to a meta-analysis by Albac et al.^5^ this form of VAP is associated with adverse clinical outcomes and an increased burden on the healthcare system. The main reason for the complexity of treatment is the ability of *A. baumannii* to rapidly develop resistance to most known antibiotics. An analytical report by the global consortium Antimicrobial Resistance Collaborators^6^ shows that carbapenem resistance among A. baumannii isolates in a number of regions, especially in Asia, reaches 60–80%, which significantly complicates the clinical management of such patients. High levels of resistance are directly associated with increased mortality in VAP, from 20% to almost 50%, as evidenced by Alnimr.^7^

A review of the current literature shows that, despite the relatively low level of global resistance to colistin, about 7% worldwide and 6.9% in the United States, its systemic use is often limited by significant nephrotoxicity, which, according to Yang and Wei,^8^ is observed in up to 36% of patients. In this regard, inhaled colistin is considered an alternative approach, which, as indicated by Alnimr,^7^ is associated with less severe kidney damage. However, the overall clinical efficacy of nebulizer therapy remains a subject of scientific debate. Studies conducted by Ungthammakhun et al.^9^ and Hao et al.^10^ demonstrate the positive pharmacokinetic properties of inhaled colistin, in particular its ability to achieve high local concentrations in the lung parenchyma.

This study fills in the knowledge gap on the clinical effectiveness of inhaled colistin for the treatment of VAP brought on by *Acinetobacter baumannii*. Although nebulised colistin has encouraging pharmacokinetic advantages, its therapeutic efficacy in comparison to systemic treatment is yet unknown, especially with regard to lowering nephrotoxicity. The objective of the study was to evaluate the therapeutic effects of intravenous versus inhalation antibiotic treatment in *Acinetobacter* VAP, specifically assessing infection control, respiratory function, and organ dysfunction in critically ill patients.

## 2. MATERIALS AND METHODS

This prospective, multicenter, randomized, double-blind interventional trial included 202 patients with traumatic brain injury (TBI) who developed VAP and sepsis. Conducted at four Ukrainian medical institutions (the Departments of Anesthesiology and Intensive Care of Kyiv City Hospital No. 4 (Kyiv), Ternopil Regional Hospital (Ternopil), Khmelnytsky Regional Hospital (Khmelnytsky), and Kyiv Clinic “Ryering” (Kyiv)) from February 1, 2023, to March 1, 2024, the study was part of research project “Improving existing and newly developed methods of prevention, diagnosis and treatment of the most common and socially significant diseases”, registered under state number 0119U002307 and affiliated with Ternopil National Medical University. As all participants were unconscious due to the severity of their condition, informed consent for participation was obtained from their legally authorized representatives. This procedure is in accordance with Ukrainian legal regulations.

All 202 patients enrolled in the study fulfilled the inclusion criteria and did not meet any of the exclusion criteria. Stratified random sampling was used, with patients allocated as follows: 50 patients from Kyiv City Hospital No. 4, 55 from Ternopil Regional Hospital, 47 from Khmelnytsky Regional Hospital, and 50 from Kyiv Clinic “Ryering.” Stratification was based on TBI severity, sepsis, and VAP to ensure proportional representation and balance across hospitals.

### 2.1. Inclusion criteria

Severe TBI confirmed by clinical and imaging data, neurosurgical intervention, pressure-controlled mechanical ventilation ≥48 hours, and VAP diagnosed ≥48 hours post-intubation with progressive infiltrates on serial chest X-rays and positive *A. baumannii* culture from tracheal/bronchial aspirates.^11^ Patients with severe TBI were included because this condition commonly accompanies and predisposes to VAP in ICU settings; it reflects the real clinical profile of critically ill, mechanically ventilated patients and, therefore, ensures that the study population corresponds to typical VAP cases. Exclusion criteria: age <18 years, baseline sepsis, renal failure, other pathogens present, colistin or amoxicillin-sulbactam resistance, no clinical response after 72 hours, drug allergies, pulmonary comorbidities affecting diagnosis, decompensated chronic illness, and life expectancy <24 hours.

The sample size of 202 patients was calculated using Cochran’s formula for a randomized clinical trial with two independent groups. The calculation was based on a 95% confidence interval (z = 1.96), 80% power (β = 0.2), and a 5% error margin (Type I error). It was expected that there would be a minimum clinically significant difference of 10% between the two treatment groups. Based on earlier research, the effect size was predicted to be 0.5.

$$n=\frac{2\times {\left(z_{\frac{\alpha }{2}}+z_{\beta }\right)}^{2}\times \sigma^{2}}{d^{2}},$$ (1)

where *n* represents the required sample size per group, $z_{\frac{\alpha }{2}}$ is the z-score for the chosen confidence level (1.96 for 95% CI), *z_β_* is the z-score for the desired power (0.84 for 80% power), σ^2^ represents the population variance, and d is the expected difference between the two groups. Using these parameters, the calculated total sample size was 202 patients. This ensures 80% power to detect a significant difference at the 5% level of significance.

Computer-generated random allocation was used to allocate patients to treatment groups, resulting in two similar cohorts: 102 patients in the intravenous (IV) group and 100 patients in the inhalation group. The randomisation process made sure that baseline attributes remained balanced even though the final distribution was not numerically equal.

Colistin methanesulfonate was given to the IV group in three separate doses totalling 9 million IU/day.^12^ Using a vibrating-mesh nebuliser, the inhalation group was given 4 million IU of nebulised colistin every 12 hours.^12^ These dosage schedules allow for a realistic evaluation of the therapeutic effects of each administration route and are consistent with current recommendations for managing VAP.

All hospitals involved in the study used colistin from the same manufacturer to ensure consistency in treatment and minimise potential variations in drug efficacy due to differences in formulation.

### 2.2. Operational definitions

In this study, several key parameters were defined to assess the therapeutic effects of antibiotic treatment in patients with VAP. Two standardised clinical scoring systems were used to evaluate organ dysfunction and infection severity. Primary endpoints were 28-day mortality and treatment efficacy through clinical pulmonary infection score (CPIS) on Day-14.^13^ The CPIS is a composite indicator that includes body temperature, leukocyte count, tracheal secretion features, oxygenation (PaO_2_/FiO_2_ ratio), chest radiography findings, and microbiological culture results to aid in the diagnosis and follow-up of VAP. A reduction of ≥2 points in CPIS from baseline to Day-14 was considered a significant improvement in infection control.^13^

Secondary endpoints included acute kidney injury incidence, sepsis, and sequential organ failure assessment (SOFA) scores on day 14.^14^ The SOFA score evaluates respiratory, cardiovascular, hepatic, coagulation, renal, and neurological characteristics to quantify the extent of multi-organ dysfunction. Greater systemic organ damage and a more severe lung infection are indicated by higher SOFA and CPIS values, respectively.^13,14^ Pre-treatment (Leukocyte 1, Procalcitonin 1, PaO_2_/FiO_2_1, CPIS1, SOFA1) and Day-14 indicators (Leukocyte 2, Procalcitonin 2, PaO_2_/FiO_2_, CPIS2, SOFA2) were recorded.^15^

Statistical analysis used Easy R (EZR) v.1.64^16^ with Shapiro-Wilk, Mann-Whitney U, Wilcoxon, Fisher’s exact, and chi-square tests; logistic regression identified predictors of sepsis and mortality (α = 0.05).

Severe TBI was diagnosed per Fourth Edition Guidelines^17^ as a blunt or penetrating injury to the head resulting in a Glasgow Coma Scale^18^ score of 3–8, confirmed by clinical and imaging data, and requiring neurosurgical intervention.

All patients received prophylactic low molecular weight heparin, standardized supportive therapy including intravenous electrolytes, enteral nutrition via nasogastric tube, glycemic control (8–11 mmol/L), and protein intake ≥2 g/kg/day (primarily B. Braun products) during the 28-day observation period.

The data collection instruments included routine clinical assessments and laboratory tests, along with imaging techniques and biomarkers, all collected through standard medical protocols during patient treatment and follow-up.

The trial adhered to the ethical principles outlined in the Declaration of Helsinki and received formal approval from the Bioethics Committee of Ternopil National Medical University (Decision No. 256, dated December 14, 2022). The study was registered in the Ukrainian Clinical Trial Registry (registry number: UCT-2023-01) on January 5, 2023.

## 3. RESULTS

Baseline demographic and clinical data in the study groups are demonstrated in Table 1. Evaluating these indicators, we can say that they did not differ from each other, except body mass index (BMI) (*p* < 0.049) (it was higher in the second group). Table 1 shows no statistically significant differences in age (*p* = 0.187), sex distribution (18% vs. 18.6% female, *p* > 0.999), or initial Glasgow Coma Scale scores (*p* = 0.244). The predominance of Glasgow Coma Scale (GCS) scores of 8 (80% and 83.3%, respectively) confirmed uniform severe TBI severity at baseline. BMI differed significantly (*p* = 0.049): median 28.7 (IQR: 27.55–30.1) in the inhalation group versus 27.9 (IQR: 26.7–29.9) in the intravenous group.

Aside from BMI, groups were well-matched at baseline using robust statistical methods (Mann-Whitney U, Fisher’s exact/Chi-square tests), permitting fair outcome assessment. The high proportion of severe TBI patients (GCS ≤ 8) reflects clinical complexity and the need for optimized antimicrobial strategies. Balanced age (median 45 and 47 years) and sex ratios minimized demographic bias. Treatment significantly normalized leukocyte counts and CPIS scores in both groups but did not change PaO_2_/FiO_2_ ratios. Procalcitonin levels increased post-treatment with inhaled colistin. SOFA scores and creatinine worsened in both groups (Table 2).

Both nebulized and intravenous colistin significantly reduced leukocyte counts and CPIS scores (Table 2), indicating improved infection control. Leukocyte normalization was more pronounced with intravenous administration (median 7.8·10^9^/L vs. 8.7·10^9^/L, *p* = 0.003), suggesting greater systemic anti-inflammatory effect. CPIS scores decreased from 8 to 2 in both groups (*p* < 0.001), with no post-treatment difference (*p* = 0.943), indicating comparable VAP resolution efficacy. PaO_2_/FiO_2_ ratio improved slightly in the inhalation group (337–343 mmHg) but remained unchanged with intravenous therapy (*p* = 0.010). SOFA scores worsened in both groups, with greater deterioration in the inhalation group (12–14) versus the intravenous group (12–13, *p* < 0.001),

Sepsis and mortality outcomes showed no significant route-related differences (Table 3). Sepsis occurred in 33% with inhaled versus 22.5% with intravenous colistin (*p* = 0.116); 28-day mortality was 27% versus 20.6% (*p* = 0.362).

Univariate logistic regression analysis examined treatment effects on outcomes, assessing the influence of treatment type, age, sex, BMI, Glasgow Coma Scale scores, CPIS, SOFA, PaO_2_/FiO_2_ ratio, leukocyte count, procalcitonin, and creatinine levels (pre- and post-treatment) on sepsis and death risks (Table 3). No association was found between pre-treatment indicators and sepsis or death risk (*p* > 0.05 in all cases, except sex in mortality analysis). Post-treatment indicators significantly influenced sepsis and death risk: PaO_2_/FiO_2_, leukocyte count, blood creatinine level, and CPIS and SOFA scores.

It was found that the gender of the patient had a significant influence on the risk of death: men had a significantly greater chance of dying, *p* < 0.046, OR = 0.44 (95% CI 0.20–0.99) (Table 4).

## 4. DISCUSSION

In the presented study, VAP was caused by *Acinetobacter baumannii*, which is currently one of the most pathogenic microorganisms spreading in intensive care units. The mortality rate for infections caused by A. baumannii is usually between 34% and 43%, and in cases of meningeal involvement, it reaches 70%.^19,20^

According to the current study, though the difference in BMI between the groups was modest, this may affect respiratory physiology and nebulized colistin deposition. High severity of brain injury and organ dysfunction may have diminished benefits of localized versus systemic administration, underscoring the need for adjunctive strategies in this high-risk cohort. All key prognostic markers emerge post-treatment, confirming the decisive role of clinical dynamics under therapy. Strongest predictors include elevated procalcitonin, leukocytosis, deteriorated PaO_2_/FiO_2_ ratio, increased creatinine, and higher CPIS and SOFA scores, reflecting the relationship between inadequate infection control, organ dysfunction, and mortality. The absence of effect from administration route confirms that overall physiological condition, not delivery method, determines prognosis.

Aerosol and intravenous colistin produced different clinical effects but did not affect sepsis incidence or 28-day mortality. Despite improved respiratory index and creatinine in the inhalation group, overall condition (SOFA scores) favored intravenous treatment. Univariate analysis showed neither therapy reduced sepsis nor death risk. Complication risk was determined by post-treatment parameters: respiratory index, leukocyte count, procalcitonin, creatinine, CPIS, and SOFA scores. Thus, VAP antibiotic therapy effectiveness depends on post-treatment patient condition, not merely pharmacological characteristics.

*A. baumannii* belongs to the so-called ESKAPE group – six microorganisms that have a high level of virulence and the potential to develop antibiotic resistance. The abbreviation ESKAPE stands for Enterococcus faecium, Staphylococcus aureus, Klebsiella pneumoniae, Acinetobacter baumannii, Pseudomonas aeruginosa, and Enterobacter spp.^4^ This list is important in a global context, as carbapenem-resistant A. baumannii has been added to the World Health Organization (WHO) list of critically important pathogens due to the global threat of antibiotic resistance.^21^

The most serious problem associated with *A. baumannii* is its ability to develop multiple and pan-resistance. As noted in a study by Seymour et al.^22^ and Kalil et al.^23^ this pathogen implements a wide range of defense mechanisms, including changing targets for antibiotic action, producing β-lactamase enzymes, modifying porins, and actively removing drugs from the cell. According to current Centers for Disease Control and Prevention (CDC) antimicrobial resistance classification criteria (updated in 2022), an *A. baumannii* culture is considered multidrug-resistant if it is resistant to at least one drug from three or more of the following classes: third- and fourth-generation cephalosporins, fluoroquinolones, aminoglycosides, carbapenems, piperacillin-tazobactam, and ampicillin-sulbactam. This framework is used to standardise multidrug-resistant (MDR) reporting and guide clinical decision-making.

Colistin is the most commonly used antibiotic for inhalation therapy in VAP, particularly that caused by multidrug-resistant strains of A. baumannii. Its physicochemical properties, including hydrophilicity, high molecular weight, and cationic nature, limit its penetration into lung tissue when administered systemically.^24^ Inhalation administration, on the other hand, ensures high drug concentrations in the lung parenchyma with minimal systemic exposure, potentially reducing nephrotoxicity. Pharmacokinetic and pharmacodynamic studies support the efficacy of inhaled colistin therapy in mechanically ventilated patients with VAP, as it achieves direct exposure to the affected area.^25,26^ According to a systematic review, the addition of inhaled colistin to intravenous therapy achieves a cumulative clinical success rate of 80% in the treatment of VAP caused by Gram-negative flora, predominantly A. baumannii.^27^

Inhaled colistin is also effective in combination with intravenous administration, as confirmed by a study evaluating intra- and extrapulmonary concentrations of the drug in critically ill patients.^28^ This combined use was associated with a reduction in 30-day mortality.^29^ However, not all authors have found advantages to combination therapy. In a study comparing inhaled plus intravenous colistin with intravenous colistin monotherapy, no statistically significant difference in clinical outcomes was found.^30^ Aerosol antibiotic therapy is considered an alternative to systemic administration due to its localized action directly in the respiratory tract. The high local concentration of the drug in the lung tissue contributes to improved bacterial clearance while reducing systemic toxicity, which is especially important for severely ill patients with impaired renal function.

Despite promising pharmacokinetic and pharmacodynamic data, aerosolized antibiotic therapy remains underutilized in clinical practice. According to Ehrmann et al.^26^ in an observational study involving 80 intensive care units, only 5% of physicians reported routine use of nebulized antibiotics. Nonetheless, this method is generally well tolerated by mechanically ventilated patients and allows for localized drug delivery. Inhalation therapy ensures high pulmonary concentrations, although deposition is frequently heterogeneous and mostly limited to proximal airways, with approximately 15% of the administered dose reaching the lungs.^31^

The effectiveness of inhalation depends significantly on the choice of delivery device. Mesh nebulizers, such as the Aerogen Solo model used in this study, provide more uniform and effective aerosolization than traditional jet nebulizers. The use of this advanced device may partially account for differences in outcomes compared to other studies. However, challenges in optimizing inhalation protocols and the limited availability of robust clinical data hinder broader implementation.^26^ There exists a noticeable gap between experimental evidence supporting the utility of inhaled antibiotics and the lack of conclusive clinical trials confirming their safety and efficacy. Systematic reviews by Zhang et al.^32^ and Valachis et al.^33^ emphasize this inconsistency, underlining the need for more large-scale randomized trials to validate the clinical benefits of nebulized colistin in VAP.

### 4.1. Clinical recommendations

The results of this study suggest that for the treatment of VAP in critically ill patients with severe traumatic brain injury, both intravenous and inhaled colistin therapies should be taken into consideration. While intravenous colistin showed superior systemic anti-inflammatory effects and preserved organ function, inhaled colistin showed improved respiratory function with decreased nephrotoxicity. Therefore, depending on the specific conditions of each patient, clinicians may decide to use these treatments; for patients who are more likely to experience nephrotoxicity, inhaled colistin is recommended. To maximise treatment results, additional research into combined therapies may be taken into consideration.

### 4.2. Limitations of the study

The study is limited by its single-country sample, modest cohort size, and lack of external validation. Results might have also been impacted by variations in clinical care amongst centres.

## 5. CONCLUSION

Inhaled colistin in VAP patients with severe TBI improved respiratory function and reduced nephrotoxicity, while intravenous administration decreased inflammatory markers and SOFA scores. Inclusion of TBI patients was clinically relevant because this group has a high incidence of VAP due to prolonged ventilation and severe systemic compromise. However, 28-day mortality and sepsis rates showed no significant differences between routes. Post-treatment clinical indicators, such as respiratory index, leukocyte count, procalcitonin, CPIS, and SOFA scores, were the primary determinants of outcomes, not the administration route itself. This emphasizes the importance of monitoring overall patient condition rather than focusing solely on the antibiotic delivery method in critically ill VAP patients.

## ACKNOWLEDGMENTS

The author(s) have no acknowledgements to declare.

## AUTHORS’ CONTRIBUTIONS

KK, OO, and MM: Conceptualization, methodology, Data curation, Writing-original draft preparation. SY: Visualization, Investigation, and Supervision. AD: Software, Validation, Writing-Reviewing, and Editing. All authors read and approved the final manuscript.

## CONFLICT OF INTERESTS

The authors declare that there is no conflict of interest.

## DISCLOSURE OF AI

The author(s) declare that generative AI-assisted tools were not used in any way to prepare, write, or complete this manuscript.

## DATA AVAILABILITY

The data that support the findings of this study are available on request from the corresponding author.

## FUNDING

This research did not receive any specific grant from the public, commercial, or not-for-profit funding agencies.

## ETHICAL APPROVAL

The trial adhered to the ethical principles outlined in the Declaration of Helsinki and received formal approval from the Bioethics Committee of Ternopil National Medical University (Decision No. 256, dated December 14, 2022). Conducted at four Ukrainian medical institutions from February 1, 2023, to March 1, 2024, the study was part of research project 0119U002307.

## INFORMED CONSENT

As all participants were unconscious due to the severity of their condition, informed consent for participation was obtained from their legally authorized representatives.

## Figures and Tables

**Table 1.** Basic demographic data and indicators characterizing the general condition of patients before treatment: women and interquartile range.

Index		Inhalation (*n* = 100)	IV (*n* = 102)	*p*
Age (years)		45 (39.0–54.0)	47 (43–55)	0.187
BMI (units)		28.7 (27.550–30.1)	27.9 (26.7–29.9)	0.049
Sex (f)		18 (18%)	19 (18.6%)	>0.999
GLASGOW (points)	5	1 (1%)	0 (0%)	0.244
	6	3 (3%)	0 (0%)	
	7	16 (16%)	17 (16.7%)	
	8	80 (80%)	85 (83.3%)	

Note: To compare quantitative characteristics, the Mann-Whitney test was used; for qualitative characteristics, Fisher’s exact test or the Chi-square test was used.

**Table 2.** Median and interquartile range of some clinical indicators at the end of treatment.

Index	Inhalation (*n* = 100)		IV (*n* = 102)		*p*
	before	after	before	after	
PaO_2_/FiO_2_, mm Hg	337 (322–354)	343 (311.5–354)	334 (322–344)	334 (321–342)	0.010
Leuc • 10^9^/L	12.4 (11.9–12.8)	8.7 (7.7–13.9)*	12.4 (11.9–12.8)	7.8 (6.9–9.4)*	0.003
Procal, ng/ml	0.7 (0.65–0.8)	0.8 (0.7–2.3)*	0.7 (0.7–0.8)	0.7 (0.6–0.9)	<0.001
CPIS, points	8 (8-8)	2 (2–2)*	8 (8–8)	2 (2–2)*	0.943
SOFA, points	12 (11.5–12)	14 (12–14)*	12 (12–12)	12 (12–13)*	<0.001
Creat, mmol/l	94.5 (88–104)	119.5 (102–142.5)*	89 (86–102)	124 (112–136)*	0.010

Note: *Denotes difference from the indicators before treatment, *p* < 0.001; p represents the difference between the corresponding indicators in different groups after treatment; to compare indicators in groups, the Mann-Whitney test was used; for comparison with indicators in one group before and after treatment, the Wilcoxon T-test was used.

**Table 3.** Univariate analysis of the risk of developing sepsis in logistic regression models.

Independent variables		Coefficients of model, b ± m	The level of significance of the difference of the coefficient from 0, *p*	OR (95% CI)
treatment	Inhalation	Reference		
	IV	−0.53 ± 0.32	0.099	0.59 (0.32–1.10)
Age, per year		−0.009 ± 0.013	0.500	0.99 (0.97–1.02)
Sex	f	Reference		
	m	−0.50 ± 0.34	0.131	0.56 (0.26–1.19)
BMI, per kg/m^2^		−0.00 ± 0.08	0.993	1.00 (0.86–1.17)
Glasgo		−0.34 ± 0.31	0.271	0.71 (0.38–1.31)
PaO_2_/FiO_2_, per 10 mm Hg,		0.001 ± 0.008	0.901	1.00 (0.98–1.02)
Leuc1, per 10^9^/L,		0.05 ± 0.22	0.834	1.05 (0.68–1.60)
Procal1, per ng/ml,		−0.97 ± 1.43	0.498	0.38 (0.02–6.26)
CPIS1, per 1 Un,		−0.08 ± 0.12	0.507	0.92 (0.73–1.17)
SOFA1, per 1 Un,		−0.12 ± 0.24	0.626	0.89 (0.56–1.42)
Creat1, per mmol/l,		0.001 ± 0.010	0.906	1.00 (0.98–1.02)
PaO2/FiO22, per 10 mm Hg,		−0.17 ± 0.03	<0.001	0.84 (0.79–0.90)
Leuc2, per 10^9^/L,		1.95 ± 0.49	<0.001	7.00 (2.65–18.49)
Procal2, per ng/ml,		11.58 ± 6.18	0.061	11.104 (0.58–20.109)
CPIS2, per 1 Un,		2.60 ± 0.39	<0.001	13.42 (6.30–28.56)
SOFA2, per 1 Un,		All with Sepsis have a score >13		
Creat2, per mmol/l		0.24 ± 0.04	<0.001	1.27 (1.18–1.37)

**Table 4.** Univariate analysis of the risk of death in logistic regression models.

Independent variables		Coefficients of model, b ± m	The level of significance of the difference of the coefficient from 0, *p*	OR (95% CI)
treatment	Inhalation	Reference		
	IV	−0.58 ± 0.36	0.102	0.56 (0.28–1.12)
Age, per year		−0.019 ± 0.015	0.219	0.98 (0.95–1.01)
Sex	f	Reference		
	m	−0.81 ± 0.41	0.046	0.44 (0.20–0.99)
BMI, per kg/m^2^		−0.015 ± 0.088	0.864	1.02 (0.85–1.21)
Glasgo		−0.53 ± 0.33	0.108	0.59 (0.31–1.12)
PaO2/FiO21, per 10 mm Hg,		−0.003 ± 0.009	0.747	1.00 (0.98–1.02)
Leuc1, per 10^9^/L,		−0.01 ± 0.24	0.981	0.99 (0.62–1.60)
Procal1, per ng/ml,		−1.20 ± 1.61	0.456	0.30 (0.01–7.08)
CPIS1, per 1 Un,		−0.12 ± 0.13	0.371	0.89 (0.68–1.15)
SOFA1, per 1 Un,		−0.25 ± 0.27	0.346	0.78 (0.46–1.31)
Creat1, per mmol/l,		−0.007 ± 0.011	0.547	0.99 (0.97–1.02)
PaO2/FiO22, per 10 mm Hg,		−0.077 ± 0.011	<0.001	0.93 (0.91–0.95)
Leuc2, per 10^9^/L,		0.93 ± 0.15	<0.001	2.53 (1.88–3.41)
Procal2, per ng/ml,		2.99 ± 0.40	<0.001	19.87 (9.02–43.79)
CPIS2, per 1 Un,		1.76 ± 0.23	<0.001	5.82 (3.70–9.16)
SOFA2, per 1 Un,		All deaths have a score >13		
Creat2, per mmol/l		0.12 ± 0.02	<0.001	1.14 (1.10–1.19)
